# Tracking of wisent–bison–yak mitochondrial evolution

**DOI:** 10.1007/s13353-012-0090-4

**Published:** 2012-03-14

**Authors:** Joanna Zeyland, Łukasz Wolko, Daniel Lipiński, Anna Woźniak, Agnieszka Nowak, Marlena Szalata, Jan Bocianowski, Ryszard Słomski

**Affiliations:** 1 Department of Biochemistry and Biotechnology, Poznan University of Life Sciences, Wołynska 35, 60-637 Poznan, Poland; 2 Institute of Human Genetics, Polish Academy of Sciences, Strzeszynska 32, 60-479 Poznan, Poland; 3 Department of Mathematical and Statistical Methods, Poznan University of Life Sciences, Wojska Polskiego 28, 60-637 Poznan, Poland

**Keywords:** *Bovini*, Wisent, Phylogeny

## Abstract

One of the most informative sources which allow the drawing of far-reaching conclusions about the origins and phylogenetics of many species, including domestic animals and humans, is mitochondrial DNA (mtDNA). One of the important research targets should include the identification of similarities between wild and domestic species. The analysis involved the nucleotide sequences of mtDNA of wisent, auroch, bison, yak, bovine reference sequence (BRS) T3, T3a, T3b, T1, T1a, T1’2’3, T2, T3, T4, T5, Q, Q1, P, R, I1, and I2 bovine haplotypes. The non-coding D-loop regions were excluded from the evolutionary analysis and 15,419-bp coding sequences were used in the final dataset. Trees constructed on the basis of whole mitochondrial genomes or on total mtDNA coding sequences alignment were generally in agreement with previous studies on the *Bovini* tribe. American bison shows stronger maternal relationships to yak than to wisent. It seems that the isolation and divergence of wisent took place early, almost 2 to 1.6 million years ago. This appears to be compatible with the paleontological date, indicating Late Pleistocene speciation of *Bison bonasus*. The yak/bison mitochondrial transfer model is in agreement with our mutation analysis and phylogenetic tree. The bison/yak mutations were collected in the bison mitochondrial genome before the transfer. After the transfer, the parallel accumulation of unique mutations took place. According to our assessment, the transfer took place at about 700 ky. The characteristic feature of the wisent and bison evolution is the maintenance of mtDNA variability, despite the fact that both species underwent population bottlenecks. Our studies did not reveal any impact of these phenomena populations in the analyzed mitochondrial genomes.

**Electronic supplementary material:**

The online version of this article (doi:10.1007/s13353-012-0090-4) contains supplementary material, which is available to authorized users.

## Introduction

The purpose of molecular phylogenetic analysis is to reconstruct evolutionary events on the basis of the analysis of nucleotide or amino acid sequences. One of the most informative sources which allow the drawing of far-reaching conclusions about the origins and phylogenetics of many species, including domestic animals and humans, is mitochondrial DNA (mtDNA) (Bruford et al. 2003; Cavalli-Sforza and Feldman 2003). Variants of mtDNA are inherited as a whole and the accumulation of mutations is the only source of variability. Due to the mentioned mtDNA properties, an evolutionary analysis is easier, as it allows the treatment of a set of polymorphic sites in mtDNA, i.e., its haplotype, as an entity. This provides the basis for the possibility of assessment of the time when a given new type, race, or species became established and, in particular, what the phylogenetic consanguinity of the examined animals is. Regions of considerable sequence variability are situated in the mtDNA non-coding segment, i.e., the so-called D-loop. Mammalian mtDNA is characterized by a lack of introns, presence of one copy of orthologic genes, absence of recombination events, and a high level of mutations (Pesole et al. 1999).

*Bovini* is one of the tribes exerting a strong influence on human civilization. This tribe had a considerable economic, cultural, and agricultural effect in both historical and contemporary societies. All contemporary domesticated cattle species appear to derive from the extinct wild aurochs (*Bos primigenius*) (Loftus et al. 1994). Fossil material, derived from the Tertiary, found in the Northern India, made it possible to conclude that aurochs originated from India, from where they spread widely. Following a period of rapid expansion, first due to climatic changes and, later, to man’s increasing farming activities—including animal pasture grazing as well as intensifying hunting and poaching—the areas occupied by aurochs began to shrink gradually.

Several decades ago, a strong selection of cattle individuals of desirable phenotypic traits increased very significantly, leading to a considerable improvement of production yields but, at the same time, not enough attention was given to genetic diversity. The modern selection methods successfully increase production, but they simultaneously result in a dramatic loss of genetic variability. On the other hand, territorial expansion of domestic cattle has resulted in an increased intentional or accidental threat of domestic cattle DNA introgression into the wild populations’ genomes, leading to phylogenetic confusions (Groves 1981; Bergthorsson et al. 2003).

Several parts of the *Bovini* phylogeny have not yet been resolved. The mitochondrial genes analysis revealed anomalous divergence between American and European bison (Janecek et al. 1996; Schreiber et al. 1999; Ward et al. 1999). On the basis of mitochondrial genes analysis, Verkaar et al. (2004) found clause clustering between yak and American bison and the anomalous position of the wisent in the phylogeny reconstruction. However, the results of the Y-chromosomal genes analysis and further molecular markers studies were consistent with the obvious phenotypic similarity and cross-fertility of the two bison species (Buntjer et al. 2002; Verkaar et al. 2004; Decker et al. 2009; MacEachern et al. 2009). In many organisms, phylogenetic trees based on nuclear and cytoplasmic (chloroplast or mitochondrial) markers indicate dissimilar relationships among the studied species. This cytoplasmic–nuclear incongruence could be explained by introgression, horizontal gene transfer, androgenesis, or errors in a phylogenetic reconstruction (Riesenberg et al. 1996; Cathey et al. 1998; Sullivan et al. 2004; Chan and Levin 2005; Fehrer et al. 2007; Linnen and Farrell 2007; Hedtke and Hillis 2011).

In this study, we present the complete mitochondrial genome of wisent (NC_014044) (*Bison bonasus*) and use it to reconstruct the phylogenetic relationships between wisent (European Bison), bison (*Bison bison*), and yak (*Bos grunniens*).

## Materials and methods

### Samples

The experimental material consisted of the peripheral blood of European Bison (a kind gift from Wrocław Zoo, Poland). Total DNA was extracted from blood using the guanidium/isothiocyanate method, as described by Ciulla et al. (1988).

### Polymerase chain reaction amplification and DNA sequencing

Mitochondrial genome sequences of wisent were amplified utilizing sets of 41 primers overlapping the whole mtDNA (see Table 2 of the supplementary material). Primers were designed using the *Bos taurus* mtDNA reference sequence (GenBank V00654). Polymerase chain reaction (PCR) was conducted in a Veriti Thermal Cycler (Applied Biosystems) in 25-μl reactions containing 125 ng total genomic DNA, 1× ReadyMix™ (Sigma Aldrich), and 12.5 μM of each primer. The PCR amplification profile consisted of an initial denaturation at 94°C for 5 min, followed by 30 cycles at 94°C for 45 s, 56°C for 45 s, 72°C for 90 s, and a final extension at 72°C for 10 min. PCR products were purified by agarose gel electrophoresis and QIAquick isolation (Qiagen). The PCR fragments were ligated directly into the StrataClone™ PCR Cloning Kit (Stratagene, Agilent Technologies). The *Escherichia coli* competent cells were transformed with the recombinant plasmid vectors. Positive clones checked by colony PCR were selected and DNA was sequenced bi-directionally using automated genetic analyzers (Applied Biosystems Prism). All fragments were sequenced several times. The number of sequencing repeats depended on the observed frequency of differences with other sequences (4–15 repeats).

### DNA sequence analysis

DNA sequences were analyzed using ChromasPro software (Technelysium Pty Ltd.). The locations of protein coding and rRNA, tRNA genes were checked through BLAST comparisons of GenBank sequences from domesticated cattle (Bovine Genome Sequencing and Analysis Consortium et al. 2009).

### Phylogenetic analysis

**Fig. 1:**
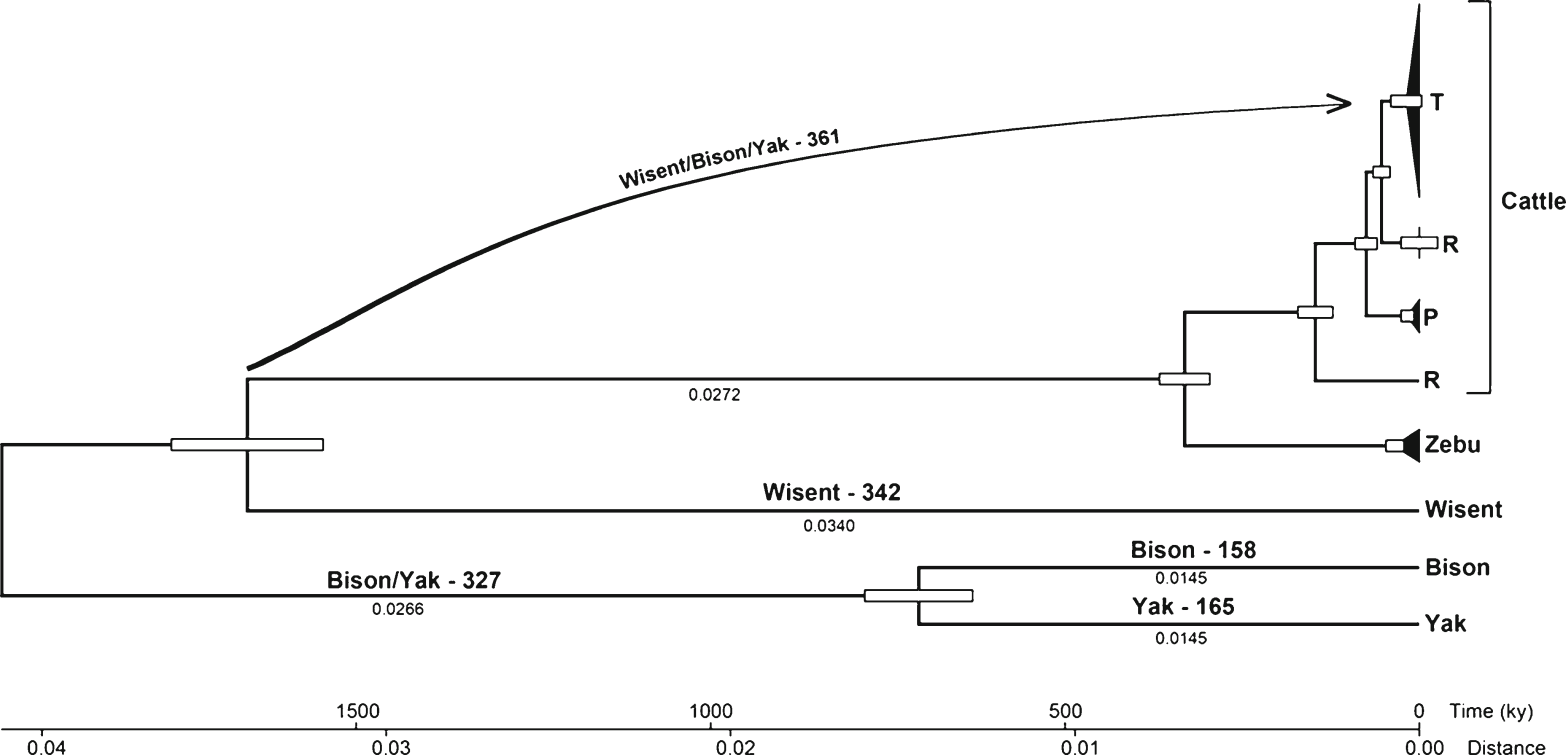
Phylogenetic tree of the representatives of *Bovini* (cattle haplogroups, zebu, wisent, bison, and yak) based on coding mitochondrial DNA (mtDNA) sequences. Unique and shared mutation numbers were assigned to appropriate branches. The values of branch length are shown below lines and error bars demonstrate the standard error after 1,000 bootstrap replicates

The analysis involved 19 nucleotide sequences of mtDNA (see Table 3 of the supplementary material). The non coding D-loop regions were excluded from the evolutionary analysis and 15,419-bp coding sequences were used in the final dataset. The sequence alignments were computed using MEGA 5.1 with the ClustalW method (Tamura et al. 2007). PhyML 3.0 software was used for the maximum likelihood evolution analysis reconstruction of the phylogenetic tree based on the general time reversible model (GTR) (Tavaré 1986; Guindon and Gascuel 2003). The phylogenetic tree was constructed and tree branches were tested by the approximate likelihood-ratio test (aLRT) (Anisimova and Gascuel 2006). The maximum likelihood test of the molecular clock hypothesis for a given tree topology and sequence alignment were performed using MEGA 5.1. The error bars on the tree branches (Fig. 1) illustrate standard errors estimated by a bootstrap procedure (1,000 replicates) in the computing of pairwise distances between sequences. Analyses were conducted using the Tamura–Nei model (Tamura and Nei 1993). The rate variation among sites was modeled with a gamma distribution (shape parameter = 0.1).

The uncertainty of molecular clock dating was estimated with the Bayesian approach by computing with the program BEAST 1.4.6 (Drummond and Rambaut 2007), which offers two statistical distributions for describing changes in rate across a branch (Drummond et al. 2006); rates can be drawn independently from either a log-normal distribution (UCLN) or an exponential distribution (UCED). To find out which distribution fits the present data best, we initially fixed the tree topology to topology consensus (T_C_). The data were partitioned, with each partition assigned a GTR + I + G substitution model. BEAST Markov chain Monte Carlo (MCMC) steps of 25 × 10^6^ generations following a burn-in of 10^5^ generations were performed for UCLN, UCED, and CLOCK models. We calculated the Bayes factor by comparing harmonic mean model likelihoods and used it to choose between models. For both non-autocorrelated models, we also calculated the covariance among branch rates, which indicated the degree of ancestor–descendant autocorrelation of rates across the tree. Using the optimal model, we then accommodated topology by removing the restriction of a fixed tree. Three replicate runs of 25 × 10^6^ generations were performed to check for convergence of the MCMC. Mean parameter estimates and 95% highest posterior densities (HPDs) were determined through analyzing the combined BEAST tree files in TreeAnnotator 1.4.6. We refer to these results with topology flexible (T_F_).

## Results

The evolutionary history was inferred by using the maximum likelihood method. The highest likelihood logarithm (lnL) obtained was estimated at −30,380, gamma shape parameter: 0.867, Ts/Tv ratio: 17.07. Bayesian analysis in Multidivtime delivered positive but very small values for the degree of autocorrelation of substitution rates across both topologies (Table 1). Finally, the analysis of T_C_ using BEAST indicated that non-autocorrelated models of rate variation fit the data significantly better than a molecular clock (Bayes factor equal to 17.73, *p* < 0.001) (Table 2). Of the non-autocorrelated models, the log-normal distribution (UCLN) had a much better harmonic mean model likelihood than the exponential distribution (UCED), and relaxation (T_F_) of a fixed topology further indicated fit. Using each of these uncorrelated models, the covariance of substitution rates between ancestor and descendant branches across the tree was not significantly different from zero.

**Table 1:** Degree of autocorrelation in rates of molecular evolution by partition and tree T_C_ topology

Genetic partition	Autocorrelation (95% confidence interval)
First positions	0.00188 (0.00132, 0.00311)
Second positions	0.00447 (0.00221, 0.00701)
Third positions	0.00490 (0.00257, 0.00735)

**Table 2:** Model comparison for analyses relaxing the assumption of autocorrelation of rates across the tree. Harmonic mean model likelihoods were calculated from post-burn-in Markov chain Monte Carlo samples generation in BEAST. For these model comparisons, the topology was fixed as T_C_. The strict clock model serves as a base comparison. The tree T_F_ refers to analyses where the topology is not fixed. Covariance indicates the degree of substitution rate autocorrelation between ancestor and descendent branches

Model	Model likelihood	Covariance
T_C_		
CLOCK	−30,395	N/A
UCED	−29,954	0.031 (−0.112, 0.195)
UCLN	−27,459	0.077 (−0.034, 0.203)
T_F_		
UCLN	−26,901	0.054 (−0.066, 0.184)

The phylogenetic tree shown in Fig. 1 illustrates the hypothetic order of evolutionary events and evolutionary relationships between cattle, wisent, bison, and yak. The wisent shows the coding region’s sequence divergence to the bison and yak at a level 6.3%. There is only a 2.6% difference between the bison and the yak sequences. Wisent, bison, and yak show, respectively, 5.3%, 6.1%, and 6.4% differences to *Bos taurus* (bovine reference sequence [BRS] V00654). There were a total of 15,419 positions of full coding sequences (without D-loops) of the mitochondrial genomes of *Bison bison* (EU177871) and BRS haplotype T3. Achilli et al. (2008) reported a *Bison bison*–*Bos taurus* sequence divergence value of 7.8%, but this was not their own result. They cited Verkaar et al. (2004) and Hassanin and Ropiquet (2004), and both articles were based just on fragments of mtDNA.

To estimate the divergence time, we assumed a constant rate of mtDNA mutation and calibrated the molecular clock based on the bison/yak–cattle bifurcation time as 2 million years (fossil records following Achilli et al. 2008). At this condition, the evolutionary rate was estimated (2.05 × 10^−8^ base substitution per nucleotide per year) by converting the substitution distances from the phylogenetic tree into time.

Due to the relationships between wisent, bison, and yak, we carried out a manual analysis of the mutations in the aligned sequences. The mutations are described in Table 4 of the supplementary material. The BRS mtDNA sequence served as the artificial root, so mutations were identified in comparison to the BRS. Manual analysis allowed identification of the oligonucleotide micro-reorganization, which is likely to represent a single mutation event, while software alignment may interpret them falsely as a range of substitutions (e.g., wisent 1472–1479).

The speciation of the common ancestor of bison and yak versus cattle was estimated (based on fossil records) as 2 million years ago, while the final separation of bison and yak species took place at only about 706 ky ago (Fig. 1). The divergence of wisent and cattle ancestors occurred 1.65 million years ago (Fig. 1). The unique mutations were accumulated after speciation of the taxa. The *Bison bonasus* (342 mutations) haplotype contains over two times more unique mutations compared with *Bison bison* (158) or *Bos grunniens* (165), so the speciation of wisent should have taken at least twice as long as bison and yak (Fig. 1, see Table 4 of the supplementary material). The bison/yak shared mutations should have been accumulated in mtDNA of their common ancestor during the period between wisent separation and final bison–yak bifurcation (or mitochondrion transfer). The wisent/bison/yak shared mutations could be a reflection of the mutation process in mtDNA during *Bos taurus* development after the wisent separation. Similar mutation numbers of wisent and wisent/bison/yak may support this hypothesis (Fig. 1).

## Discussion and conclusions

*Bison bonasus* (Linnaeus 1758, European bison, wisent) together with American bison are classified under the genus *Bison* and there is no fertility barrier between this species. However, an unexpected divergence of the mitochondrial genes from American and European bison has been reported (Janecek et al. 1996; Schreiber et al. 1999; Ward et al. 1999; Verkaar et al. 2004). Morphological studies (Groves 1981; Geraads 1992) and comparison of mitochondrial sequence analysis suggest a clustering of yak with bison (Pitra et al. 1997; Schreiber et al. 1999; Verkaar et al. 2004). However, nuclear genome studies did not support bison/yak mitochondrial relationships (Buntjer et al. 2002, Decker et al. 2009; MacEachern et al. 2009).

Several explanations have been proposed for the mitochondrial divergence of the bison species (Janecek et al. 1996; Verkaar et al. 2004). The phenotypic convergence is unlikely because of the wisent and bison amplified fragment length polymorphism (AFLP) patterns. The hypothesis of the reduction of the evolution rate could be falsified by statistical tests (Janecek et al. 1996).

Verkaar et al. (2004) proposed lineage sorting during *Bos* and *Bison* speciation. According to this hypothesis, the isolation of bison/yak mtDNA took place early and then the split of zebu/taurine and wisent lines occurred. Next, the repeated introgression events between Eurasian cattle female and bison male ancestors led to the creation of wisent with mtDNA from maternal ancestors. Our analysis of whole mitochondrial genomes makes this model rather unlikely because of the high number of shared wisent/bison/yak mutations. This hypothesis has not even explained the observed bison/yak mtDNA similarity. The last possible explanation is interspecific hybridization, which seems to be very attractive in the same aspects of bison/yak mitochondrial speciation.

In many organisms, phylogenetic trees based on nuclear and cytoplasmic (chloroplasts of mitochondrial) markers indicate different relationships among the studied species (Riesenberg et al. 1996; Cathey et al. 1998; Sullivan et al. 2004; Chan and Levin 2005; Fehrer et al. 2007; Linnen and Farrell 2007; Hedtke and Hillis 2011). Trees constructed by the authors using whole mitochondrial genomes or total mtDNA coding sequences alignment were generally in agreement with previous studies. American bison shows stronger maternal relationships to yak than to wisent. It seems that the isolation and divergence of wisent took place early, almost 2–1.6 million years ago. This appears compatible with paleontological data, indicating Late Pleistocene speciation of *Bison bonasus* (Nielsen-Marsh et al. 2002; Verkaar et al. 2004).

If bison/yak mtDNA similarity reflects the real evolution homology of these species, the bifurcation of bison and yak has taken place at about 700 ky. Recently, Bibi and Vrba (2010) tried to draw attention to incomplete or speculative characteristics of *Bovini* fossil data. However, phenotypic differences and the nuclear genome studies make the model of close bison/yak relationship unlikely (Buntjer et al. 2002; Decker et al. 2009; MacEachern et al. 2009).

More feasible seems the hypothesis where interspecific hybridization and subsequent backcrossing generated transfer of the mitochondrial genome between two species. After the transfer event, the acceptor species would be shaped by the founder effect and population bottlenecks. The transfer from yak to bison seems to be more probable because this would explain both the close relationships between bison and yak, and the bison/wisent relationship’s incongruity. However, the mitochondrial control region from the bones of the extinct *Bison priscus* (steppe wisent) that lived in Eurasia earlier seems to be more related to *Bison bison* than to *Bison bonasus* (Nielsen-Marsh et al. 2002; Verkaar et al. 2004), and that may support the opposite direction of genetic transfer.

Thulin and Tegelström (2002) described the process of interspecific hybridization between brown hare introduced in Sweden with the native mountain hare, leading to a transfer of the mitochondrion. Interestingly, only the transfer of the mountain to brown hare was observed. They proposed that frequency-dependent hybridization and interspecific male competition mediates this directionality. Similar mechanisms may have played a role during bison/yak hybridization.

The yak/bison mitochondrial transfer hypothesis is in agreement with our mutation analysis and phylogenetic tree. The bison/yak mutations were collected in bison mitochondrial genome before transfer. After the transfer, parallel accumulation of unique mutations took place. According to our assessment, the transfer took place at about 700 ky.

The characteristic feature of the wisent and bison evolution is the maintenance of mtDNA variability, despite the fact that both species have undergone recent bottlenecks because both species were threatened with extinction. Our studies did not reveal any impact of these population phenomena in the analyzed mitochondrial genomes.

There are just two complete sequences of wisent mtDNA available at this moment in the GenBank database, HQ223450.1 and NC_014044. The comparative analysis revealed 52 differences, including 38 substitutions and 14 indels mutations. Most differences (46) gather in five islands inside the mentioned genes. The largest one includes 16 substitutions and 4 indels (20) and can be found in the 16S rRNA gene, between 1,471 and 1,693 nt. The next one is localized in the NADH2 gene and contains four substitutions in a range of only 50 nucleotides (4,931–4,980). The substitution 4980 A > G results in the amino acid change Ala > Ile. Further differences are found in the NADH3 gene, where ten synonymous substitutions occur between 108 nucleotides (9,858–9,966). In the NADH4 gene, there are eight non-synonymous substitutions, including seven indels and one substitution (10,898–10,949). There are two stretches of amino acid sequences that appear only in *Bison bonasus* HQ223450.1 sequences (in NADH4: VNTQVPNTLLLCTPDGGTKQ, and in CYTB: ETTAEF, caused by insertions which introduced frameshifts). The introduction of such long stretches is surprising, considering the high conservation of mitochondrial proteins.

European bison as the species was driven almost to extinction and is classified by the International Union for Conservation of Nature (IUCN) as a vulnerable species. Wisent is one of the biggest and most impressive wild animals living in Europe, but is still poorly genetically characterized. This is caused mainly by difficult access to the research material from wisent individuals living under protection as well as from among the wildlife. This is the first published analysis of the full wisent mitochondrial genome and the first attempt of phylogenetic reconstruction considering the whole mitochondrial genome. It seems necessary to obtain access to a larger number of the samples in order to obtain more genetic information about *Bison bonasus* (e.g., haplotype diversity).

## Electronic supplementary material

Below is the link to the electronic supplementary material.

ESM 1 (DOCX 36 kb)
