# Supplementary material for: Tracking of wisent–bison–yak mitochondrial evolution
Source: J Appl Genet. 2012 Mar 14;53(3):317–22. doi: 10.1007/s13353-012-0090-4 (PMC3402669; doi:10.1007/s13353-012-0090-4)
Supplement: Supplementary file 1 — (DOCX 36 kb) [file 13353_2012_90_MOESM1_ESM.docx]

Supplemental data:

Table 2. Overlapping the whole mtDNA sets of 42 primers used to amplify mitochondrial genome sequences.

| **Primer** | **PCR product size (bp)** | **mtDNA localization**  **(nt)** |
| --- | --- | --- |
| MT-1F | 499 | 16233-393 |
| MT-1R |  |  |
| MT-2F | 449 | 15847-16295 |
| MT-2R |  |  |
| MT-3F | 494 | 15468-15961 |
| MT-3R |  |  |
| MT-4F | 466 | 15084-15549 |
| MT-4R |  |  |
| MT-5F | 432 | 14750-15182 |
| MT-5R |  |  |
| MT-6F | 447 | 14371-14817 |
| MT-6R |  |  |
| MT-7F | 400 | 14064-14463 |
| MT-7R |  |  |
| MT-8F | 400 | 13757-14156 |
| MT-8R |  |  |
| MT-9F | 495 | 13321-13815 |
| MT-9R |  |  |
| MT-10F | 494 | 12890-13383 |
| MT-10R |  |  |
| MT-11F | 488 | 12498-12985 |
| MT-11R |  |  |
| MT-12F | 479 | 12080-12558 |
| MT-12R |  |  |
| MT-13F | 462 | 11676-12137 |
| MT-13R |  |  |
| MT-14F | 496 | 11245-11740 |
| MT-14R |  |  |
| MT-15F | 483 | 10898-11380 |
| MT-15R |  |  |
| MT-16F | 445 | 10510-10954 |
| MT-16R |  |  |
| MT-17F | 447 | 10269-10715 |
| MT-17R |  |  |
| MT-18F | 400 | 9968-10370 |
| MT-18R |  |  |
| MT-19F | 488 | 9558-10047 |
| MT-19R |  |  |
| MT-20F | 461 | 9151-9612 |
| MT-20R |  |  |
| MT-21F | 470 | 8732-9201 |
| MT-21R |  |  |
| MT-22F | 487 | 8309-8796 |
| MT-22R |  |  |
| MT-23F | 498 | 7901-8398 |
| MT-23R |  |  |
| MT-24F | 577 | 7398-7974 |
| MT-24R |  |  |
| MT-25F | 535 | 6999-7533 |
| MT-25R |  |  |
| MT-26F | 499 | 6572-7070 |
| MT-26R |  |  |
| MT-27F | 485 | 6151-6637 |
| MT-27R |  |  |
| MT-28F | 497 | 5711-6207 |
| MT-28R |  |  |
| MT-29F | 498 | 5277-5774 |
| MT-29R |  |  |
| MT-30F | 400 | 4931-5331 |
| MT-30R |  |  |
| MT-31F | 598 | 4407-5004 |
| MT-31R |  |  |
| MT-32F | 481 | 3990-4470 |
| MT-32R |  |  |
| MT-33F | 478 | 3570-4048 |
| MT-33R |  |  |
| MT-34F | 499 | 3138-3636 |
| MT-34R |  |  |
| MT-35F | 495 | 2694-3189 |
| MT-35R |  |  |
| MT-36F | 480 | 2264-2743 |
| MT-36R |  |  |
| MT-37F | 500 | 1817-2316 |
| MT-37R |  |  |
| MT-38F | 475 | 1397-1873 |
| MT-38R |  |  |
| MT-39F | 491 | 991-1482 |
| MT-39R |  |  |
| MT-40F | 493 | 565-1057 |
| MT-40R |  |  |
| MT-41F | 599 | 25-624 |
| MT-41R |  |  |

Supplemental data: Table 3. List of GeneBank IDs and given haplotypes of mtDNA sequences analyzed.

|  | GenBank ID | Haplotype |
| --- | --- | --- |
|  | V00654 | Bovine reference sequence (BRS) –T3 |
|  | AY676866 | T3b |
|  | DQ124418 | T3a |
|  | EU177842 | T1 |
|  | EU177843 | T1a |
|  | EU177840 | T1’2’3 |
|  | DQ124383 | T2 |
|  | DQ124372 | T4 |
|  | EU177862 | T5 |
|  | FJ971081 | Q |
|  | EU177867 | Q1 |
|  | DQ124389 | P |
|  | FJ971084 | R |
|  | EU177868 | I1 |
|  | EU177869 | I2 |
|  | NC_014044 | *Bison bonasus* -Wisent |
|  | NC_013996 | *Bos primigenius* - Auroch |
|  | EU177871 | *Bison bison-* Bison |
|  | NC_006380 | *Bos grunniens -* Yak |

Supplemental data: Table 4. Mutations analysis. Mutations are numbered according to the BRS (Bovine reference sequence, GenBank V00654) without gaps. Suffixes indicate transitions and transversions (to A, C, G or T), indels (+, d) or range of micro-inversions or other reorganization is defined. The BRS mtDNA sequence served also as an artificial root, so mutations were identified in comparison to BRS.

| Haplotype | Number of mutation | Mutations | |
| --- | --- | --- | --- |
| Unique mutations | | | |
| Wisent (*Bison bonasus*) | 342 | 119A, 169G, 222C, 225G, 272C, 319A, 325A, 345G, 349dA, 489C, 785G, 893T, 1086C, 1170A, 1289G, 1294T, 1299T, 1318A, 1325T, 1385C, 1472-79, 1488T, 1497C, 1518C, 1601dC, 1603C, 1662C, 1665A, 1680G, 1861T, 1864T, 1872T, 1932A, 2034T, 2042C, 2078A, 2126C, 2156T, 2197T, 2206, 2501A, 2588T, 2634A, 2649A, 2653A, 2992T, 2993G, 3021C, 3039T, 3121G, 3208C, 3229C, 3270C, 3298C, 3347C, 3364T, 3404A, 3421T, 3445T, 3466C, 3523C, 3544C, 3619A, 3625T, 3643G, 3730T, 3772T, 3781A, 3790C, 3799G, 3838T, 3839T, 3910T, 3962-65, 4045G, 4050T, 4051G, 4196A, 4277C, 4282C, 4289C, 4298C, 4301T, 4322C, 4409T, 4493-96, 4577G, 4639T, 4667G, 4724G, 4808T, 4817T, 4869C, 4882C, 4922C, 4925G, 4953C, 4975T, 4988T, 5006C, 5089A, 5093T, 5144C, 5159C, 5216C, 5238C, 5357T, 5460-62,5643G, 5782C, 5812T, 5827A, 5926T, 5953C, 5965G, 5992T, 6001T, 6017T, 6070A, 6124T, 6148G, 6247T, 6262T, 6307G, 6311T, 6319G, 6334C, 6386C, 6406T, 6481G, 6538C, 6580T, 6628C, 6637T, 6643G, 6682T, 6685G, 6688A, 6739A, 6868C, 6892C, 6934C, 6988C, 7063T, 7070T, 7174T, 7177G, 7180T, 7192C, 7222T, 7256T, 7281G, 7303G, 7427G, 7454A, 7532G, 7571T, 7619G, 7623T, 7628T, 7667G, 7682A, 7703C, 8052C, 8113G, 8123A, 8262C, 8286T, 8358T, 8418G, 8436T, 8469T, 8511C, 8526C, 8553G, 8556T, 8613A, 8631C, 8679C, 8697T, 8742C, 8769G, 8811-14, 8967G, 9032C, 9134T, 9143G, 9179T, 9243T, 9254T, 9356T, 9365T, 9381C, 9419C, 9443C, 9446G, 9458G, 9485T, 9707T, 9754G, 9908C, 9987T, 10029C, 10038TC, 10072T, 10079T, 10083A, 10098A, 10110T, 10222-27, 10229C,10275C, 10355-61, 10421C, 10461T, 10561C, 10630-33, 10741A, 10816C, 10819C, 10876T, 10927C, 10939G, 10955T, 10960T, 11092T, 11176G, 11185C, 11275C, 11323T, 11344T, 11359T, 11374G, 11401G, 11434C, 11476A, 11497A, 11512C, 11617-18, 11681G, 11720T, 11770T, 11854G, 11866T, 11875G, 11959G, 11984C, 12023A, 12053C, 12121G, 12136A, 12201T, 12249T, 12270C, 12294C, 12328T, 12396T, 12462T, 12468T, 12489C, 12549T, 12565T, 12625C, 12666G, 12714T, 12729A, 12771T, 12996C, 13032C, 13134T, 13231A, 13257G, 13345C, 13383C, 13422A, 13446-49, 13503C, 13518T, 13532T, 13551C, 13596G, 13606C, 13647C, 13680T,13683T, 13695T, 13737T, 13758T, 13792T, 13794G, 13802G, 13810T, 13812T, 13830C, 13881T, 14018C, 14072T, 14077G, 14120A, 14122T, 14210C, 14219T, 14249T, 14366C, 14407C, 14448G, 14519C, 14672G, 14711C, 14714T, 14732A, 14765T, 14786C, 14855C, 14909G, 14910T, 14991C, 15047T, 15098T, 15125T, 15128T, 15213C, 15225A, 15299T, 15383G, 15416C, 15417A, 15418T, 15495T, 15566G, 15632G, 15795T, 15796dTA, 15813C, 15852T, 15860dA, 15862T 15863C, 15883C, 15918T, 15924+C, 15932T, 15934T, 15942C, 15945G, 15946C, 15960T, 15984T, 16107G, 16134G, 16200A, 16206C | |
| Bison (*Bison bison*) | 158 | 143C, 203C, 205G, 256G, 283C, 304C, 701C, 807A, 1471dA, 1627T, 1892A, 1969C, 2098G, 2146T, 2582A, 2675G, 2991G, 3181C, 3334C, 3385C, 3400T, 3559G, 3593C, 3754G, 3863C, 4048G, 4271T, 4325T, 4331T, 4382T, 4388T, 4421C, 4451C, 4454G, 4541G, 4542G, 4550C, 4628T, 4658A, 4679A, 4728T, 4734T, 4799T, 5009T, 5108T, 5224C, 5375C, 5393T, 5498C, 5803C, 5959C, 6133C, 6142G, 6181C, 6235C, 6283T, 6301T, 6322T, 6373C, 6418C, 6634A, 6661G, 6859A, 7051T, 7084G, 7297+TC, 7307A, 7382C, 7433G, 7654T, 7877G, 7952T, 8064C, 8228C, 8436G, 8512T, 8646C, 8730T, 8748C, 8766A, 8872G, 8890G, 8925C, 9185T, 9233T, 9416C, 9497G, 9644C, 9901T, 10125T, 10681C, 10689C, 10690C, 10840G, 10903G, 10924C, 11009C, 11020G, 11104C, 11164T, 11305T, 11413G, 11468A, 11651C, 11662G, 11692G, 11716G, 11722G, 11752G, 11815A, 12026C, 12195C, 12297C, 12363T, 12367G, 12399G, 12543G, 12690C, 12702T, 12727G, 12745C, 12807C, 12886T, 12963G, 13041T, 13453T, 13467C, 13594T, 13663A, 13725T, 14039T, 14042G, 14136G,14351T, 14372C, 14523C, 14558T, 14638C, 14702T, 14834T, 14990C, 15269T, 15302T, 15419C, 15422C, 15443T, 15461T, 15570A, 15706G, 15895G, 15914G, 15919T, 15941dA, 15989A, 16045T, 16048T, 16073G, 16304C | |
| Yak (*Bos grunniens*) | 165 | 292A, 378T, 379T, 418G, 726C, 740T, 968C, 987G, 1082A, 1169+T, 1193G, 1226G, 1328T, 1489T, 1718T, 2570G, 2748T, 2967T, 2981G, 3140T, 3313C, 3322G, 3377C, 3421A, 3615C, 3697C, 3827T, 3832G, 3850T, 3906T, 3955G, 3982G, 4129T, 4286C, 4364C, 4385T, 4550T, 4622G, 4634T, 4859T, 4878C, 4894C, 5087T, 5105C, 5259-67, 5288G, 5395T, 5631G, 5982C, 6019G, 6229-32, 6256C, 6565T, 6607G, 6748C, 6793T, 6799T, 6877T, 6895C, 6904T, 7015T, 7114G, 7132G, 7276C, 7367G, 7418G, 7509T, 7523A, 7565T, 7643T, 7697C, 7946G, 7991T, 8000C, 8110G, 8114G, 8332C, 8529G, 8845G, 8868T, 8871T, 8934C, 9420C, 9422G, 9518G, 9590T, 9867T, 9927T, 9945C, 10020T, 10119C, 10293T, 10343G, 10577A, 10613A, 10660A, 10714T, 10753G, 10798T, 10864T, 11153T, 11209T, 11212T, 11422T, 11581T, 11632C, 11710C, 11720A, 11764C, 11782G, 11791T, 11851T, 11878T, 12035T, 12144G, 12170C, 12207G, 12228T, 12292G, 12375C, 12528G, 12579G, 12582G, 12612T, 12738T, 12864G, 12867A, 12922T, 12945T, 13086T, 13143T, 13255G, 13503G, 13506T, 14192T, 14243C, 14522T, 14603G, 14624A, 14756T, 14801C, 14813G, 14972T, 15002G, 15014G, 15082C, 15236T, 15275C, 15386G, 15398T, 15422T, 15434G, 15440T, 15461C, 15554C, 15557T, 15656A, 15922T, 15949T, 16067G, 16108C, 16115C, 16164C, 16199G, 16308C, | |
| Shared mutations in accordance with phylogenetic tree | | |  |
| wisent/bison/yak | 361 | 201dA, 221+C, 234C, 279T, 296C, 315A, 329A, 824T, 923C, 1017G, 1083C, 1087C, 1158T, 1166C, 1308T, 1457A, 1481A, 1495A, 1499A, 1598dA, 1610C, 1694A, 1833T, 1869T, 1871T, 1879A, 1981C, 2026C, 2031T, 2039A, 2117C, 2172-73, 2185C, 2220T, 2318C, 2434A, 2553A, 2558A,2642A, 2877C, 2953T, 2977C, 2979G, 2988T, 2990A, 3051G, 3163A, 3190C, 3292C, 3325C, 3335C, 3388T, 3526T, 3535A, 3550A, 3574A, 3600T, 3637A, 3668T, 3698C, 3793C, 3805C, 3844C, 3874C,3879-83, 3892-95, 3907A, 3940C, 3985C, 3988A, 4000A, 4018A, 4139T, 4168C, 4252C, 4297T, 4316C, 4361A, 4391T, 4562A, 4625A, 4649A, 4740A, 4769A, 4823C, 4862T, 4871A, 4892T, 4913C, 4989A, 5045C, 5072A, 5084C, 5156A, 5180C, 5186T, 5272C, 5285A, , 5300A, 5500A, 5501T, 5604C, 5670A, 5731C, 5758C, 5890T, 5899G, 5962G, 6013T, 6109C, 6127C, 6269T, 6274C, 6280A, 6313G, 6314C, 6370C, 6379C, 6403G, 6427T, 6436A, 6451C, 6457C, 6460A, 6499A, 6533T, 6553T, 6568A, 6772T,7027T, 7069T, 7135A, 7219C, 7330C, 7359C, 7397C, 7448T, 7514A,7526T, 7538A, 7583T, 7586T, 7655C, 7700A, 7830C, 7851C, 7865C, 7932A, 7944C, 7976A, 8015T, 8109A, 8164A, 8188C,8204T, 8212A, 8230T, 8308G, 8326A, 8343C, 8370C, 8394T, 8478C, 8514A, 8607T, 8806T, 8870T, 9038C, 8996C, 9060T, 9088C, 9146G, 9221C, 9242C, 9269T, 9278T, 9296T, 9335T, 9386C, 9431A, 9488T, 9509G, 9563C, 9602C, 9809C, 9873T, 9978C, 10066A, 10071C,10047A, 10113G, 10134C, 10216G, 10304T, 10322T, 10349A, 10388T, 10418C, 10521C, 10576A, 10645T, 10651T, 10750C, 10691A, 10777C, 10825A, 10830T, 10867A, 10879C, 10888C,10918T, 10936G, 10988T, 11000T, 11228-30, 11287A, 11386T, 11551T, 11633T, 11785C, 11809A, 11824A, 1845G, 11870T, 12057dT, 12127T, 12234T, 12156A, 12193T, 12223A, 12273T, 12313T, 12327C, 12376G, 12429C, 12433C, 12486C, 12546T, 12597C, 12622G, 12654T, 12658C, 12672A, 12675T, 12684C, 12750C, 12728A, 12801A, 12894C, 12903G, 12927T, 12964T, 13056A, 13083A, 13173C, 13176C, 13206A, 13215T, 13350C, 13368C, 13392C, 13429A, 13455C, 13437C, 13509-12, 13521C, 13537G, 13539C, 13584C, 13623C, 13638C, 13671A, 13689A, 13732C, 13815T, 13857A, 13882C, 13909C,13955A, 13988A, 14036A, 14129A, 14098T, 14138A, 14198C, 14204G, 14255C, 14294C, 14321A, 14348C, 14387T, 14396C, 14402-05, 14411A, 14416A, 14570T, 14582T, 14609C, 14634T, 14708C, 14817C, 14897C, 14933T, 14951T, 14961C, 15059C, 15077T, 15134T, 15146C, 15167T, 15194A, 15206A, 15212A, 15227C, 15290A, 15308T, 15311C, 15326A, 15353T, 15413C, 15425T, 15495A, 15579A, 15582A, 15593T, 15605A, 15617A, 15629T, 15678C, 15698A, 15751G, 15818G, 15847T, 15854T, 15873C, 15878T, 15916C, 15921A, 15944C, 15951C, 15953A, 15957G, 15961A, 15963dATG, 16055+T, 16082A, 16084T, 16094C, 16109C, 16121A, 16122C, 16124dATCTATT, 16138C, 16142dA, 16200+A, 16228G, 16247T, 16255T, 16260T, 16264A, 16302C, | |
| bison/yak | 327 | 171-180, 255T, 268T, 327A, 458G, 554C, 649C, 709A, 739T, 756T, 760T, 817C, 909T, 1132A, 1190T, 1191T, 1459T, 1470A, 1480T, 1684C, 1716T, 1729T, 1822G, 1823T, 1948A, 1987G, 2021C, 2037G, 2077T, 2087T, 2145A, 2165T, 2208C, 2321T, 2335C, 2504T, 2700A, 2965A, 2971T, 3127T, 3160C, 3169A, 3223C, 3238C, 3271C, 3281T, 3289T, 3295G, 3328T, 3329T, 3448C, 3481T, 3551C, 3691C, 3808A, 3821C, 3826T, 3979C, 4006A, 4021G, 4071A, 4108T, 4248C, 4285C, 4293T, 4406C, 4448A, 4530T, 4592T, 4647T, 4743C, 4826G, 4853C, 4883C, 4889C, 4907T, 4943C, 4949A, 4997C, 5000C, 5039A, 5096C, 5166G, 5225A, 5237C, 5279G, 5292T, 5493T, 5505T, 5755A, 5824C, 5905T, 5938T, 5950C, 5968C, 5980C, 6049A, 6157T, 6220G, 6304T, 6352A, 6374T, 6508C, 6511A, 6637A, 6758C, 6763T, 6814C, 6937G, 6955G, 7043G, 7054G, 7081C, 7145C, 7409T, 7499C, 7520A, 7575T, 7589T, 7661G, 7668G, 7670A, 7769A, 7770T, 7805G, 7829A, 7847T, 7883G, 7892T, 7910G, 7970C, 7985T, 7994G, 8009A, 8134A, 8352T, 8398T, 8403G, 8405A, 8475C, 8476C, 8517T, 8542T, 8550G, 8656A, 8721C, 8775C, 8793T, 8817G, 8892-98, 8937T, 9020C, 9033-35, 9063G, 9071-77, 9089-90, 9119C, 9150A, 9155T, 9167T, 9299C, 9344T, 9440C, 9524C, 9626-36, 9650C, 9713T, 9770-71, 9829C, 9864T, 9879T, 9912T, 9921G, 10155G, 10251C, 10259T, 10275G, 10373T, 10433T, 10466C, 10493C, 10505C, 10514T, 10540T, 10624A, 10657T, 10666T, 10672T, 10678C, 10702T, 10747T, 10813C, 10822A, 10909A, 10954T, 11037T, 11095C, 11105A, 11158C, 11179C, 11245A, 11324T, 11338C, 11428T, 11440A, 11452T, 11473C, 11485T, 11627C, 11672A, 11746C, 11755T, 11758A, 11852T, 11857T, 11867T, 12016C, 12021T, 12057C, 12079G, 12148C, 12157G, 12173C, 12318T, 12334C, 12339T, 12493A, 12519A, 12525A, 12603T, 12624T, 12657C, 12687A, 12699T, 12721G, 12730A, 12758T, 12849T, 12897T, 12915T, 12946C, 12975G, 12984T, 13029T, 13050G, 13074C, 13146A, 13386C, 13389C, 13401C, 13419T, 13420T, 13458A, 13479G, 13485C, 13527C, 13563T, 13604C, 13620T, 13626C, 13646T, 13677C, 13698T, 13791T, 13806T, 13839T, 13878T, 13887T, 13893-96, 14009T, 14027C, 14105C, 14108C, 14161T, 14213C, 14273A, 14278T, 14287G, 14390G, 14468C, 14531A, 14561C, 14615C, 14687T, 14712G, 14780-83, 14837C, 14840C, 14909A, 14939G, 15005T, 15011T, 15038C, 15080T, 15173T, 15210T, 15239G, 15249A, 15371T, 15492A, 15494T, 15527C, 15559T, 15581T, 15590C, 15639C, 15739A, 15795G, 15829C, 15831C, 15846C, 15871A, 15881T, 15924dATACACAGA, 15937dC, 15954A, 15956G, 15962A, 15964dTG, 15995T, 16047A, 16056G, 16074A, 16110T, 16112C, 16132G, 16294C, | |
| Shared mutations in no accordance with phylogenetic tree – reversion | | | |
| wisent/yak  (w/o bison) | 44 | 281G, 1858+A, 3130C, 3382C, 5102T, 6656A, 7868A, 8245A, 8406T, 8421T, 8439C, 8838T, 9092T, 9096T, 9479G, 9545C, 10253T, 10828T, 11053A, 11248A, 12274T, 12330C, 12492A, 12555T, 12879T, 12969C, 13336T, 13434C, 13899T, 14063T, 14102C, 14628G, 14747T, 14867T, 15068C, 15157C, 15711C, 15819T, 15915G, 15948G, 15974T, 16051A, 16096T, 16204-05, | |
| wisent/ bison  (w/o yak) | 27 | 163G, 190T, 1496G, 4446C, 4722C, 5003T, 5177G, 5240C, 6289G, 7634G, 7949C, 9095A, 9176A, 9224C, 10577C, 11789C, 11858T, 13023T, 13038A, 13470T, 13710C, 13869C, 14144T, 15156A, 16050G, 16074C, 16118G, 16133C, | |
| bison/yak/ zebu (I)  (w/o wisent) | 23 | 248T, 296C, 518G, 737C, 3071C, 3439G, 4730G, 7358A, 8194T, 9005T, 10039T, 10137A, 10849C, 11134C, 11419C, 11842G, 12178C, 12513T, 12900C, 13098C, 13275, 13554A, 16119C | |
| wisent, zebu | 13 | 233C, 5743C, 5917G, 6340T, 8466C, 8503C, 10331G, 10445C, 11035T, 11266T, 13371C, 14825T, 15741T, | |
| yak/zebu | 13 | 166G, 2016C, 3136T, 3145A, 3931T, 12469T, 13005G, 13692C, 14120C, 16085C, 16117A, 16147C, 16232T | |
| bison /zebu | 10 | 8A, 1474T, 1492T, 9480A, 10621T, 12924T, 15105T, 16093G, 16113C, 16301T | |
